# Exploration of Alternative Approaches to Citron Kinase Inhibition for Glioblastoma Treatment

**DOI:** 10.3390/ijms27219678

**Published:** 2026-10-29

**Authors:** Alessia Ferraro, Letizia Lazzerini, Giorgia Iegiani, Francesca De Bacco, Carla Boccaccio, Ferdinando Di Cunto

**Affiliations:** 1 Neuroscience Institute Cavalieri Ottolenghi, 10043 Orbassano, Italy; alessia.ferraro@unito.it (A.F.); l.lazzerini@unito.it (L.L.);; 2 Department of Neuroscience “Rita Levi Montalcini”, University of Turin, 10126 Turin, Italy; 3 INOC—Istituto Oncologico Nazionale Candiolo, IRCCS, 10060 Candiolo, Italycarla.boccaccio@unito.it (C.B.); 4 Department of Oncology, University of Turin, 10043 Turin, Italy

**Keywords:** glioblastoma multiforme, citron kinase, kinase inhibitors, Lestaurtinib

## Abstract

Glioblastoma (GBM) remains a highly aggressive brain tumor with limited therapeutic options, highlighting the need to identify novel tumor vulnerabilities. Citron kinase (CITK), a protein involved in cytokinesis and genome stability, is required for the proliferation and survival of neural progenitors and has previously emerged as a potential therapeutic target in medulloblastoma (MB). Here, we investigated the effects of CITK depletion and pharmacological inhibition in two patient-derived GBM stem cell models. CITK was depleted by siRNA or pharmacologically targeted using the first selective kinase inhibitor so far described, C3TD879 (c879). We also explored the polypharmacological inhibitor Lestaurtinib. Effects on spheroid growth, cytokinesis, cytotoxicity, DNA damage-associated proteins, apoptotic markers, and cell-cycle regulators were assessed. CITK depletion partially impaired spheroid growth, slightly increased multinucleation, and led to cytotoxicity in both GBM models, while effects on DNA damage-associated proteins were cell-line dependent. Selective CITK inhibition with c879 slightly reduced spheroid growth and only partially recapitulated the cellular and molecular effects of CITK depletion. Lestaurtinib produced stronger spheroid growth inhibition and cytokinesis defects, while inducing limited cytotoxicity and heterogeneous molecular responses. In summary, the tested GBM models were shown to be moderately sensitive to CITK depletion and selective catalytic inhibition, while Lestaurtinib showed the strongest effects, which mimicked only in part the effects of CITK loss.

## 1. Introduction

Glioblastoma (GBM) is one of the most aggressive and lethal primary malignant brain tumors in adults, characterized by extensive infiltration, necrosis, and resistance to conventional therapies. GBM accounts for approximately 57% of all gliomas and 48% of primary malignant central nervous system (CNS) tumors [1]. Its incidence varies according to histological subtype, age at diagnosis, race, sex, and geographic region, with reported rates ranging from 0.59 to 3.69 cases per 100,000 individuals [2]. Despite advances in treatment, the prognosis of patients with GBM remains poor, with a 5-year relative survival rate of only 6.8% [3]. The standard of care for newly diagnosed patients consists of maximal safe surgical resection followed by radiotherapy with concomitant and adjuvant temozolomide (TMZ), according to the Stupp protocol established in 2005 [4]. The introduction of this regimen increased median overall survival to 14.6 months in the pivotal clinical trial [5].

The marked inter- and intratumoral heterogeneity of GBM, together with its intrinsic resistance to radiotherapy and chemotherapy, severely limits the effectiveness of current therapeutic strategies [6,7]. Although numerous clinical trials are investigating multimodal therapeutic approaches, including immunotherapy, EGFR and VEGF inhibitors, molecularly targeted therapies directed against cancer-associated signaling pathways, and oncolytic viruses [3,5,8], clinical outcomes remain unsatisfactory, highlighting the urgent need for more effective and tumor-selective therapeutic strategies.

In recent years, considerable effort has been devoted to identifying molecular vulnerabilities that are critical for tumor growth and survival. Therapeutic strategies targeting such vulnerabilities may improve treatment efficacy while limiting off-target toxicity by preferentially sparing normal tissues [9].

In this context, genes associated with primary congenital hereditary microcephaly (MCPH) may represent particularly attractive candidate therapeutic targets. MCPH-associated genes encode proteins that play critical roles in centrosome function, cell-cycle progression, the DNA damage response, and the maintenance of genomic stability in neural progenitor cells. Notably, despite being broadly expressed in proliferating tissues, several MCPH genes are particularly important for the proliferation and survival of neural progenitor cells [10]. Moreover, MCPH genes are highly expressed in several cancer types, suggesting that tumor cells may depend on their activity to sustain proliferation and survival [11].

Among the proteins encoded by MCPH-associated genes, Citron kinase (CITK) is a serine/threonine kinase that plays a critical role in the late stages of cell division in developing neural progenitors. CITK is required for cytokinesis, where it contributes to midbody organization and stability, promoting the proper completion of abscission [12,13]. In addition, several studies have implicated CITK in the maintenance of genome stability by promoting the repair of DNA double-strand breaks (DSBs) [14], as well as in the regulation of mitotic spindle orientation [15,16].

We previously demonstrated that CITK depletion exerts significant antitumor effects in medulloblastoma (MB) cells, both in vitro and in vivo. Specifically, across different MB subtypes, loss of CITK impairs cytokinesis and homologous recombination (HR)-mediated DNA DSBs repair, resulting in the accumulation of DNA damage, activation of the TP53 family, cell-cycle arrest, and apoptosis, and, importantly, reduced tumor growth in vivo [17,18,19]. Similar antiproliferative effects of CITK depletion have been reported in other tumor types, suggesting that CITK may represent a therapeutic vulnerability across multiple cancers [20,21,22,23,24,25].

On this basis, determining the extent to which the catalytic activity of CITK contributes to its tumor-promoting functions is critical for evaluating its potential as a pharmacological target. In MB cells, the requirement for CITK kinase activity was supported by the finding that the antiproliferative effects of CITK depletion could not be rescued by expression of a kinase-dead mutant [17]. However, studies in human organoids and mutant mice showed that kinase-inactivating mutations produce substantially milder phenotypes than complete loss of CITK [26], suggesting that kinase-independent functions may contribute to biological activity. Consistent with this possibility, the first highly selective CITK inhibitor developed to date [27] showed no detectable antiproliferative activity across a broad panel of tumor cell lines.

Nevertheless, given the selective vulnerability of neural progenitor cells to CITK loss or mutation and the molecular similarities between neural progenitors and GSC, the latter may exhibit a specific dependency on CITK. In the present study, we therefore investigated the effects of CITK depletion and selective pharmacological inhibition in a small sample of GSC. We also evaluated the effects of the polypharmacological inhibitor Lestaurtinib, which despite its low specificity towards CITK effectively suppressed MB cell growth and mimics many consequences of CITK loss [19]. Our results demonstrate that GSC may be sensitive to CITK depletion and, even more, to Lestaurtinib treatment. They also reveal substantial heterogeneity in the underlying molecular responses and important differences from those previously observed in MB cells. These findings provide insight into the limitations of CITK as a therapeutic target in GBM.

## 2. Results

### 2.1. CITK Knockdown Slightly Impairs GBM Stem Cell Growth and Cytokinesis

**Figure 1:**
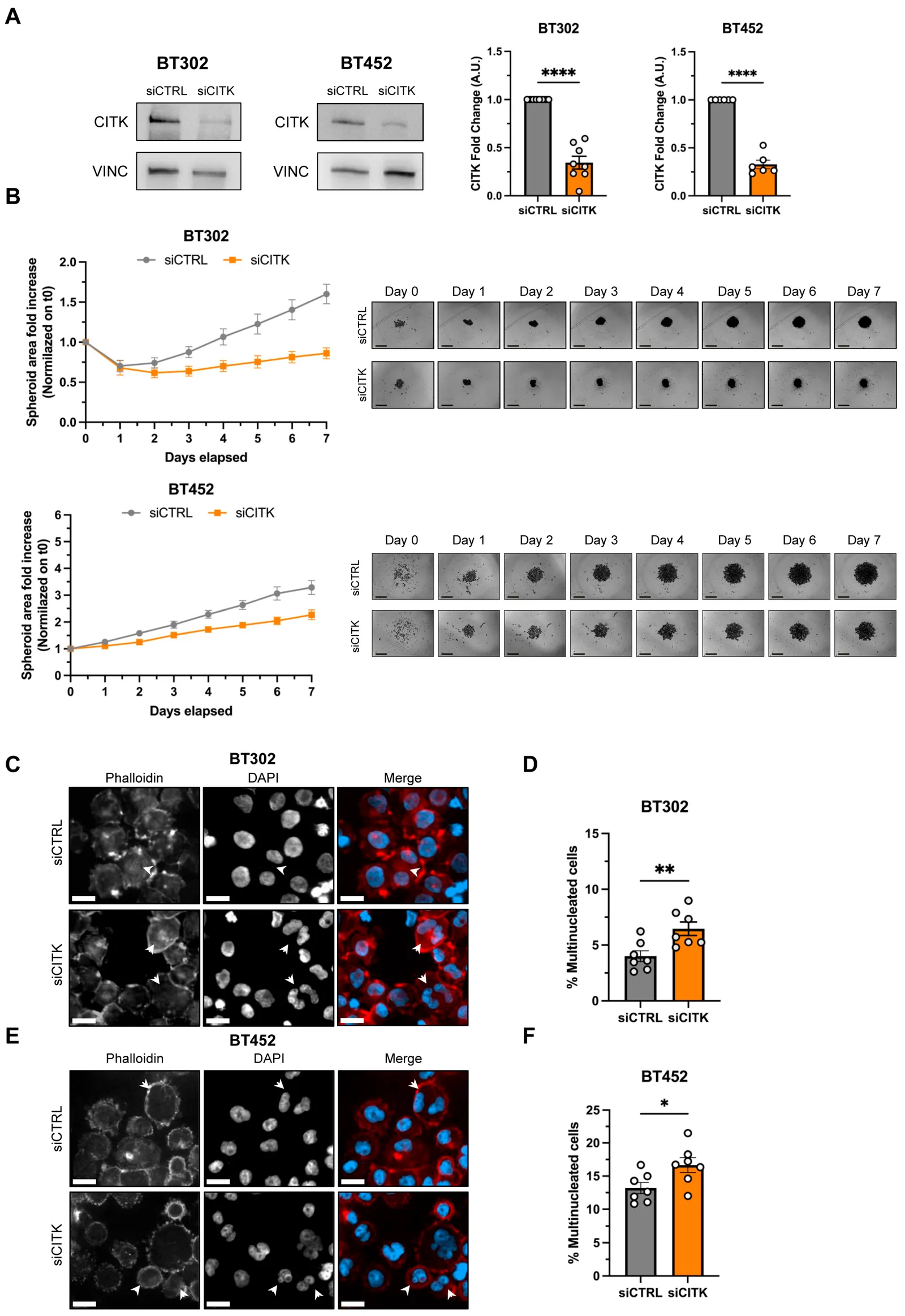
CITK knockdown in GSCs decreases spheroid growth and induces cytokinesis failure. (**A**) Western blot analysis of total lysate from the indicated GSC, 48 h after treatment, with non-targeting (siCTRL, *n* = 8) or CITK-specific siRNAs (siCITK, *n* = 6). The level of CITK was analyzed and the internal loading control was vinculin (VINC). In the panels on the right, quantification of the relative density of CITK in the indicated GSC. Each dot indicates an independent biological replicate. (**B**) Cell proliferation assay. Growth curves were obtained by assessing spheroid area, normalized on time 0, at the indicated times after treatment. *n* = 11, with each *n* consisting of three spheroids. The siCITK curves were statistically different from control curves at the following levels:BT302 *p* < 0.0001, B452 *p* < 0.0001; two-way ANOVA. Representative images of spheroids for each time point are shown. Scale bars: 800 μm. (**C**,**E**) Representative image of BT302 and BT452 cells, respectively, processed for immunofluorescence 48 h after transfection with siCTRL or siCITK and stained with DAPI (blue) and Phalloidin (red). White arrows indicate exemplar multinucleated cells. Scale bars: 20 μm. (**D**,**F**) Quantification of multinucleated BT302 and BT452 cells, respectively, in the indicated transfections, performed as in (**C**,**E**). Each dot indicates an independent biological replicate; *n* = 7. A total of >500 cells were counted for each treatment condition in each experiment. All quantifications were based on at least three independent biological replicates. Error bars, SEM. * *p* < 0.05, ** *p* < 0.01, **** *p* < 0.0001; paired two-tailed Student’s *t*-test (WB) and unpaired two-tailed Student’s *t*-test (% multinucleation). A.U., arbitrary units.

To investigate whether CITK is required for GBM cell proliferation, we used the human patient-derived GBM stem cell (GSC) lines BT302 and BT452, propagated as neurospheres, which provide clinically relevant models that retain key molecular features of the tumors of origin and recapitulate aspects of GBM heterogeneity. These GSCs had been previously molecularly and genetically characterized [6,28,29]. Both were initially classified as GBM mesenchymal subtypes according to the Verhaak gene expression signature [6,28]. Based on the latest classification of CNS tumors [30,31], the genetic characterization of these lines [29] (Supplementary Table S1) supports them as GBM, since they were both IDH wild type, mutated in TERT promoter, and had an increased EGFR copy number. In addition, although they were not directly tested for +7/−10, in both cases HGF and MET copy number was increased, CDKN2A was lost bi-allelically, and heterozygous PTEN mutations were present. Importantly, BT302 was TP53 wild type while BT452 harbored the TP53 p.G245D inactivating mutation [29] (Supplementary Table S1). Cells were transfected with CITK-specific siRNAs [13] or a non-targeting control siRNA, and knockdown efficiency was assessed by measuring CITK protein levels. CITK expression was reduced by approximately 70% in both GSCs following 48 h siRNA transfection (Figure 1A). Cell growth was then assessed using a single-spheroid assay. Cells were transfected with siRNAs at the time of seeding in ultra-low-attachment plates, with one spheroid generated per well. Spheroid growth was monitored by time-lapse imaging using the Incucyte^®^ system and quantified as the change in spheroid area over time (Figure 1B). Under control conditions, BT302 and BT452 spheroids exhibited different growth rates. By day 7 after seeding, BT302 spheroids showed a 1.6-fold increase in area, whereas BT452 spheroids showed a 3.3-fold increase (Figure 1B). CITK silencing significantly impaired spheroid growth in both GSCs. The effect was statistically significant from day 3 post-transfection in BT302 cells and from day 4 in BT452 cells. By day 7, spheroid growth was reduced by approximately 50% in BT302 cells and 32% in BT452 cells compared with the respective control conditions (Figure 1B). Since spheroid area is not necessarily proportional to cell numbers, we also performed a multi-spheroid assay, which better correlates with proliferation. Even with this test, BT302 silenced for CITK showed significantly reduced expansion compared to control 3 days after siRNA treatment (Supplementary Figure S1A). In contrast, no significant effects were measured in similarly-treated BT452 cells (Supplementary Figure S1A).

Because cytokinesis failure is one of the best-characterized consequences of CITK loss in both neural progenitors and tumor cells [13,32,33,34], we next investigated whether CITK silencing impaired cytokinesis also in GSC. At 48 h post-transfection, CITK knockdown significantly increased the proportion of multinucleated cells from 4% to 6.5% in BT302 cells and from 13% to 17% in BT452 cells (Figure 1C–F). These findings are consistent with impaired cytokinesis following CITK depletion and support a role for CITK in the proper completion of cell division in GSC cells, although the magnitude of the effect was very limited and differed between the two GSCs.

### 2.2. CITK Silencing Differentially Affects DNA Damage Response in GSC Lines

**Figure 2:**
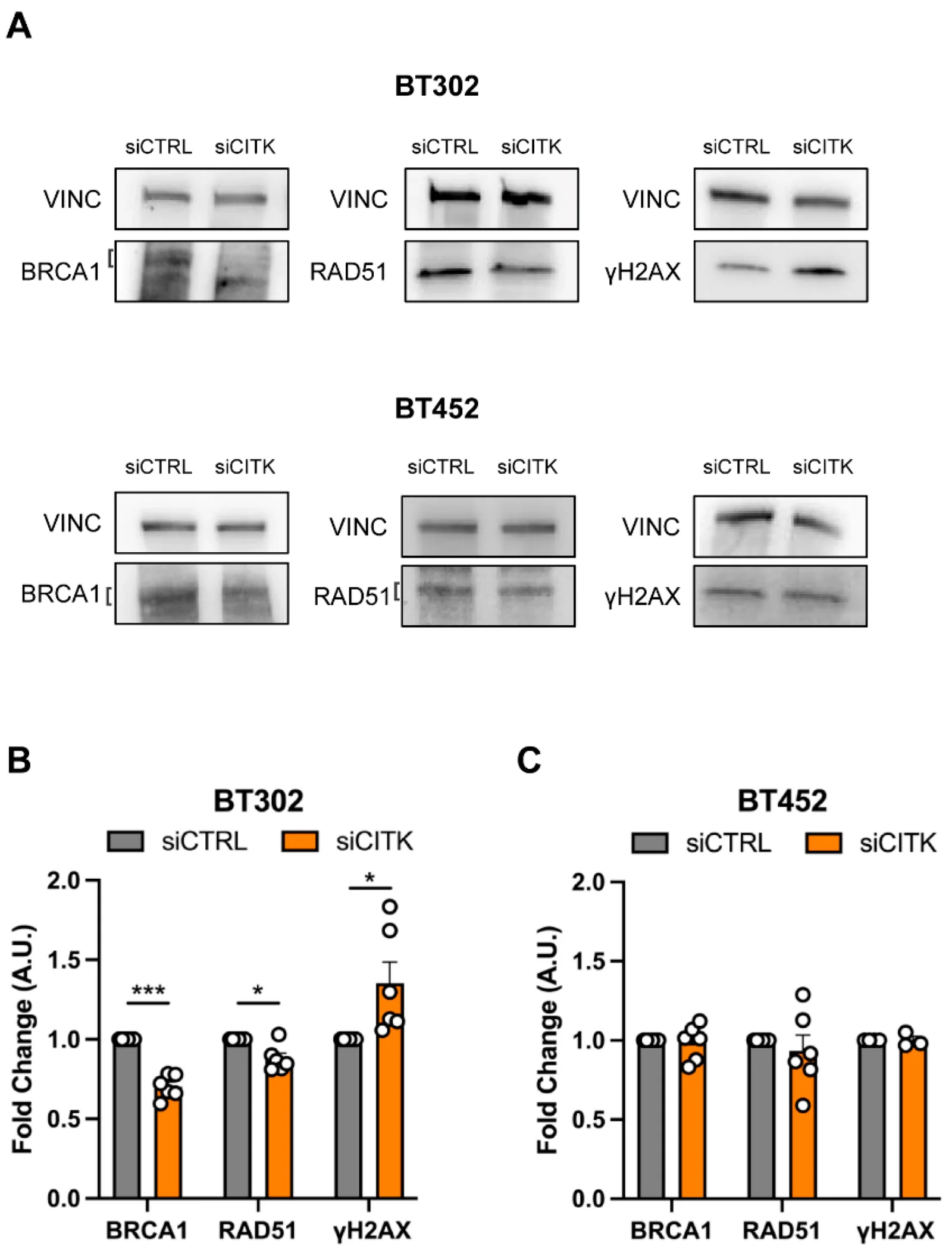
Differential effects of CITK knockdown on DNA damage and HR proteins in GSC. (**A**) Western blot analysis of total lysate from the indicated GSC, 48 h after treatment with siCTRL or siCITK. The levels of BRCA1, RAD51, and γH2AX were analyzed and the internal loading control was vinculin (VINC). For BRCA1, the signal used for quantification was the upper doublet, migrating slower than 250 kb, indicated by brackets. For RAD51, the quantified band (37 kD), is indicated by a dash. (**B**,**C**) Quantification of the relative density of BRCA1, RAD51, and γH2AX in the indicated GSC. Each dot indicates an independent biological replicate (BT302 *n* = 6; BT452 BRCA1 and RAD51 *n* = 6, γH2AX *n* = 3). Error bars, SEM. * *p* < 0.05, *** *p* < 0.001; paired two-tailed Student’s *t*-test. A.U., arbitrary units.

Previous studies have shown that CITK loss promotes the accumulation of DNA double-strand breaks (DSBs) by reducing the abundance and impairing the function of key factors involved in homologous recombination (HR)-mediated DNA repair, such as RAD51 and BRCA1 [14,18,35]. We therefore investigated the effects of CITK depletion on DNA damage markers and HR-associated proteins in GSC by Western blotting, 48 h after siRNA transfection. In BT302 cells, CITK knockdown reduced BRCA1 and RAD51 levels and increased γH2AX levels (Figure 2A,B), consistent with previous findings [14,18]. On the contrary, BRCA1, RAD51, and γH2AX levels remained largely unchanged in BT452 knockdown cells (Figure 2A,C). These findings suggest that CITK knockdown may differentially affect DNA damage signaling and HR-associated proteins across GSC.

### 2.3. CITK Depletion Increased Cytotoxicity with Limited Activation of Apoptotic Markers in GSC

**Figure 3:**
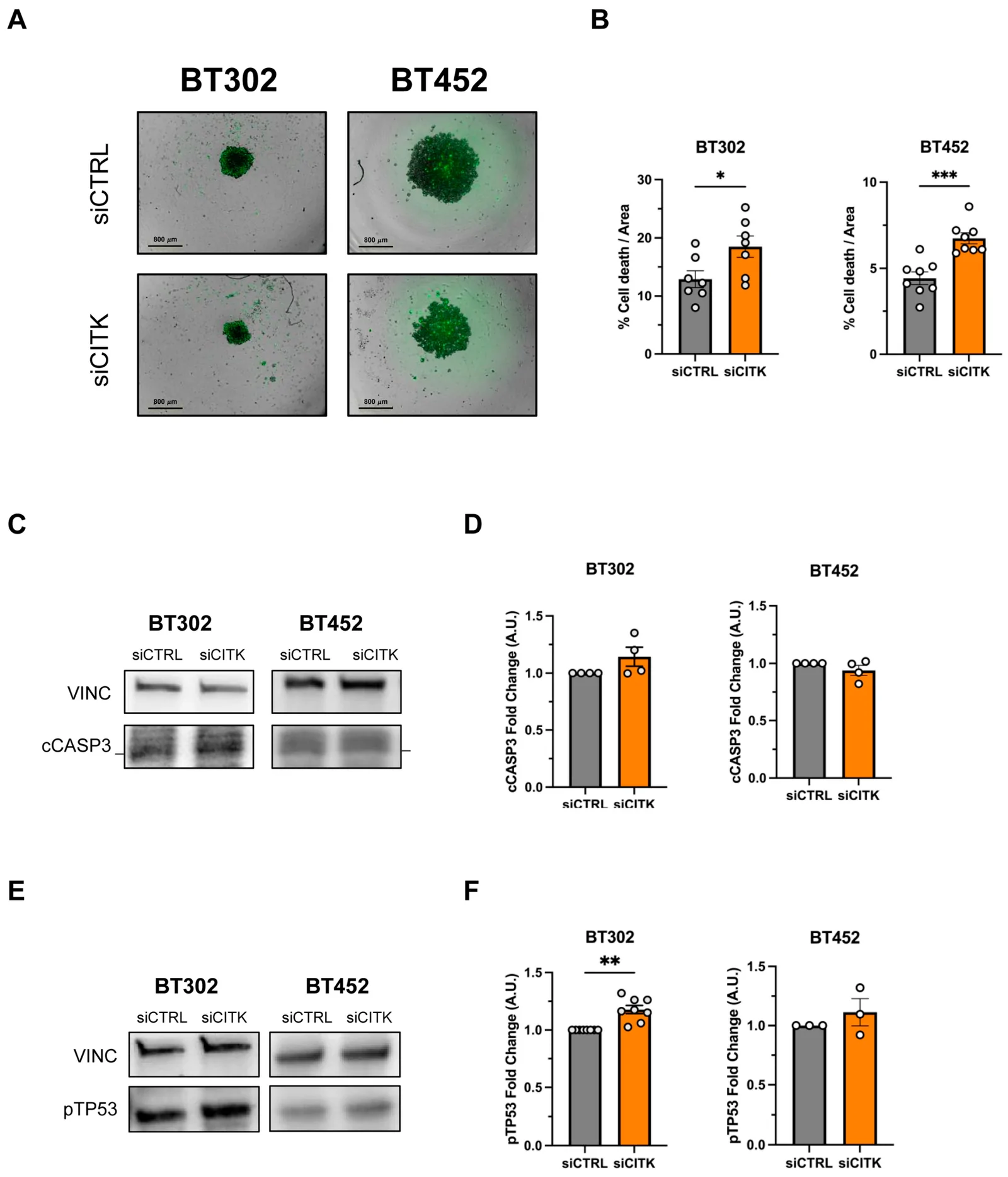
CITK knockdown has limited effects on apoptosis induction in GSC. (**A**) Representative images of BT302 and BT452 spheroids after CellTox^TM^ green cytotoxicity assay at day 7 after treatments. Scale bars: 800 μm. (**B**) Percentage of cell death quantified with green fluorescence intensity and normalized on the spheroid area of BT302 (*n* = 7) and BT452 (*n* = 8) at day 7. Each dot indicates an independent biological replicate. At least three spheroids were counted for each treatment condition in each experiment. (**C**,**E**) Western blot analysis of total lysate from the indicated GSC, 48 h after treatment with siCTRL or siCITK. The levels of cCASP3 and pTP53 were analyzed and the internal loading control was vinculin (VINC). For cCASP3, the position of the quantified band is indicated by a dash. (**D**,**F**) Quantification of the relative density of cCASP3 and pTP53 in the indicated GSC. Each dot indicates an independent biological replicate (BT302 cCAP3 *n* = 4, pTP53 *n* = 8; BT452 cCASP3 *n* = 4, pTP53 *n* = 3). Error bars, SEM. * *p* < 0.05, ** *p* < 0.01, *** *p* < 0.001; paired two-tailed Student’s *t*-test (WB) and unpaired two-tailed Student’s *t*-test (% cell death/area). A.U., arbitrary units.

To determine whether the reduction in spheroid growth following CITK depletion was associated with increased cell death, we performed the CellTox^TM^ Green cytotoxicity assay on individual GSC spheroids at day 7 post-transfection, which was the end-point of the cell growth experiment. This assay detects extracellular DNA released by cells that have lost membrane integrity [36]. CITK depletion significantly increased the cytotoxicity-associated fluorescence signal in both BT302 and BT452 spheroids (Figure 3A). Quantitative analysis showed that the CellTox-positive area accounted for 18.5% and 6.75% of the total spheroid area in CITK-depleted BT302 and BT452 spheroids, respectively, compared with 12.9% and 4.4% in the corresponding control spheroids (Figure 3B). Conversely, MTS assay revealed a significant reduction of cell viability in BT302 cells silenced for CITK, while significant differences between specific and control siRNAs were not observed in BT452 (Supplementary Figure S2A).

Looking for apoptosis induction, western blot analysis of cleaved caspase-3 (cCASP3) was performed 48 h post-transfection. Data revealed only a modest, non-significant increase in the levels of this marker in CITK-depleted BT302 cells, and no significant changes were detected in BT452 cells (Figure 3C,D). Similarly, pTP53 protein levels, which are robustly induced following CITK loss in neural progenitors and medulloblastoma models [18,35], showed a moderate increase following CITK depletion in BT302 cells and no significant variations in BT452 cells (Figure 3E,F). The limited changes in pTP53 and cCASP3 observed here suggest that canonical apoptosis is not a prominent cell death mechanism in CITK-deficient GSC lines, pointing to the possible involvement of additional cell-death mechanisms.

**Figure 4:**
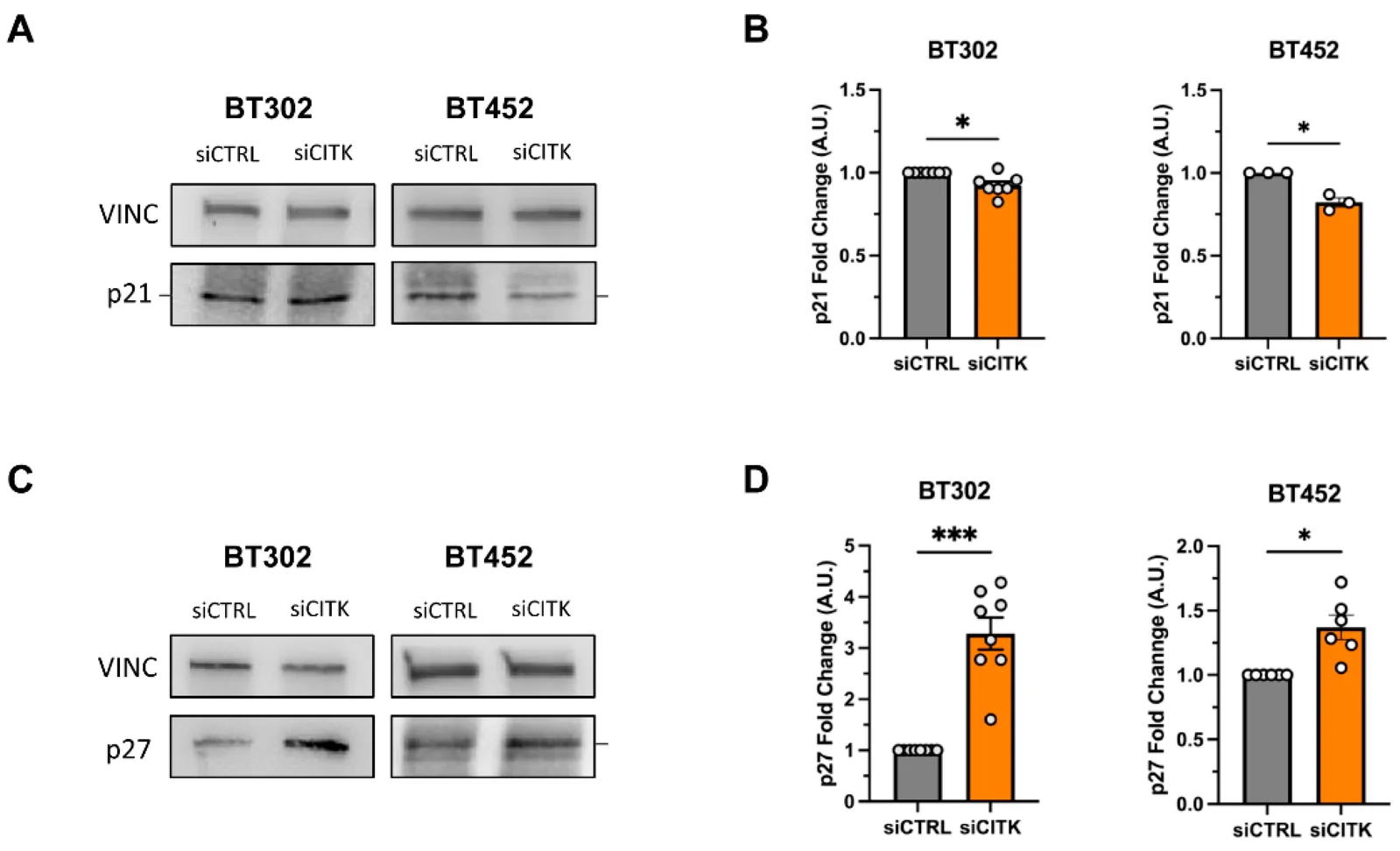
CITK knockdown has limited effects on p21 and consistently induces p27 expression in GSC. (**A**,**C**) Western blot analysis of total lysate from the indicated GSC, 48 h after treatment with siCTRL or siCITK. The levels of p21 and p27 were analyzed and the internal loading control was vinculin (VINC). Where more bands are visible, the position of quantified bands is indicated by a dash. (**B**,**D**) Quantification of the relative density of p21 and p27 in the indicated GSC. Each dot indicates an independent biological replicate (BT302 p21 *n* = 7, p27 *n* = 8; BT452 p21 *n* = 3, p27 *n* = 6). Error bars, SEM. * *p* < 0.05, *** *p* < 0.001; paired two-tailed Student’s *t*-test. A.U., arbitrary units.

We next investigated whether CITK depletion might be associated with the activation of cell-cycle arrest mechanisms, given previous evidence indicating that G1 arrest and cellular senescence may occur more frequently than apoptosis following cytokinesis failure [37]. To this end, we analyzed the protein levels of the key cell-cycle regulators p21 and p27. CITK silencing resulted in a modest but significant reduction in p21 levels in both BT302 and BT452 cells (Figure 4A,B). Conversely, p27 levels were markedly increased in both GSC, with a more pronounced effect in BT302 cells (Figure 4C,D).

Taken together, these findings indicate that, in GSC, CITK depletion may have heterogeneous effects on cell growth and cytotoxicity, while it consistently leads to p27 upregulation.

### 2.4. The Selective CITK Inhibitor C3TD879 Reduces GSC Spheroids’ Growth with Limited Effects on Cytokinesis

**Figure 5:**
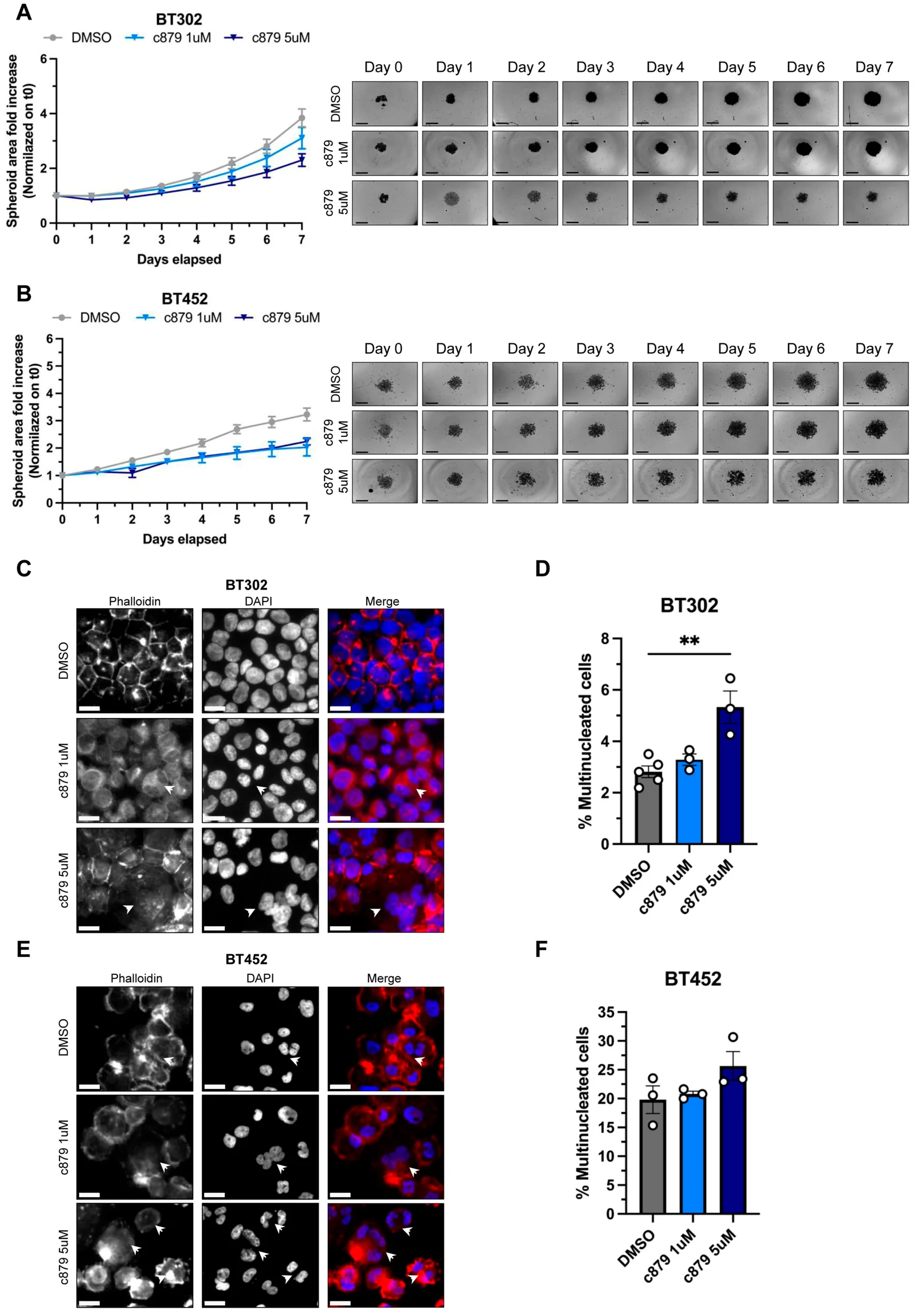
c879 decreases spheroid growth and differentially induces cytokinesis failure in GSC. (**A**,**B**) Cell proliferation assay. Growth curves were obtained by assessing the area of the spheroid, normalized on time 0, at the indicated times after treatment. 1 µM *n* = 6, 5 µM *n* = 8, with each *n* consisting of three spheroids. The treatment curves were statistically different from control curves at the following levels: BT302 1 µ M *p* < 0.05 and 5 µ M *p* < 0.0001; BT452 1 µ M and 5 µM *p* < 0.0001; two-way ANOVA. Representative images of spheroids are shown in the lower panels. Scale bars: 800 µm. (**C**,**E**) Representative image of BT302 and BT452 cells, respectively processed for immunofluorescence 48 h after DMSO, 1µM or 5µM c879 treatment and stained with DAPI (blue) and Phalloidin (red). White arrows indicate exemplar multinucleated cells. Scale bars: 20 μm. (**D**,**F**) Quantification of multinucleated BT302 and BT452 cells, respectively, in the indicated transfections, performed as in (**C**,**E**). Each dot indicates an independent biological replicate (*n* = 3). A total of >200 cells were counted for each treatment condition in each experiment. Error bars, SEM. ** *p* < 0.01; unpaired two-tailed Student’s *t*-test.

The development of C3TD879 (c879), the first highly selective CITK inhibitor, was recently reported [27]. We therefore investigated the effects of this compound on GSC. Spheroid growth was first assessed using single-spheroid assays at two c879 concentrations (1 and 5 µM). In BT302 cells, treatment with 1 µM c879 significantly reduced spheroid growth, and this effect was further enhanced at 5 µM, indicating a dose-dependent response (Figure 5A). In BT452 cells, c879 also significantly reduced spheroid growth; however, the magnitude of the effect was similar at 1 and 5 µM (Figure 5B). A significant reduction in spheroid growth was also obtained in BT452 cells in multi-spheroid assay with c879 at 5 µM (Supplementary Figure S1C). Conversely, in this case no statistically significant differences could be shown between inhibitors and control in BT302 cells (Supplementary Figure S1C).

To determine whether the CITK inhibitor may also affect cytokinesis, we quantified multinucleated cells by immunofluorescence analysis. Treatment with 1 µM c879 resulted in a modest, non-significant increase in multinucleation in both GSC. At 5 µM, c879 significantly increased the proportion of multinucleated BT302 cells (Figure 5C,D). A similar increase was observed in BT452 cells following treatment with 5 µM c879, although the effect did not reach statistical significance (Figure 5E,F). Overall, these results indicate that selective pharmacological inhibition of CITK impairs GSC spheroid growth, while producing relatively modest effects on cytokinesis under the tested conditions.

### 2.5. C879 Increased Cytotoxicity, Does Not Markedly Affect DNA Damage-Associated Markers and Differentially Modulates P27 in GSC

**Figure 6:**
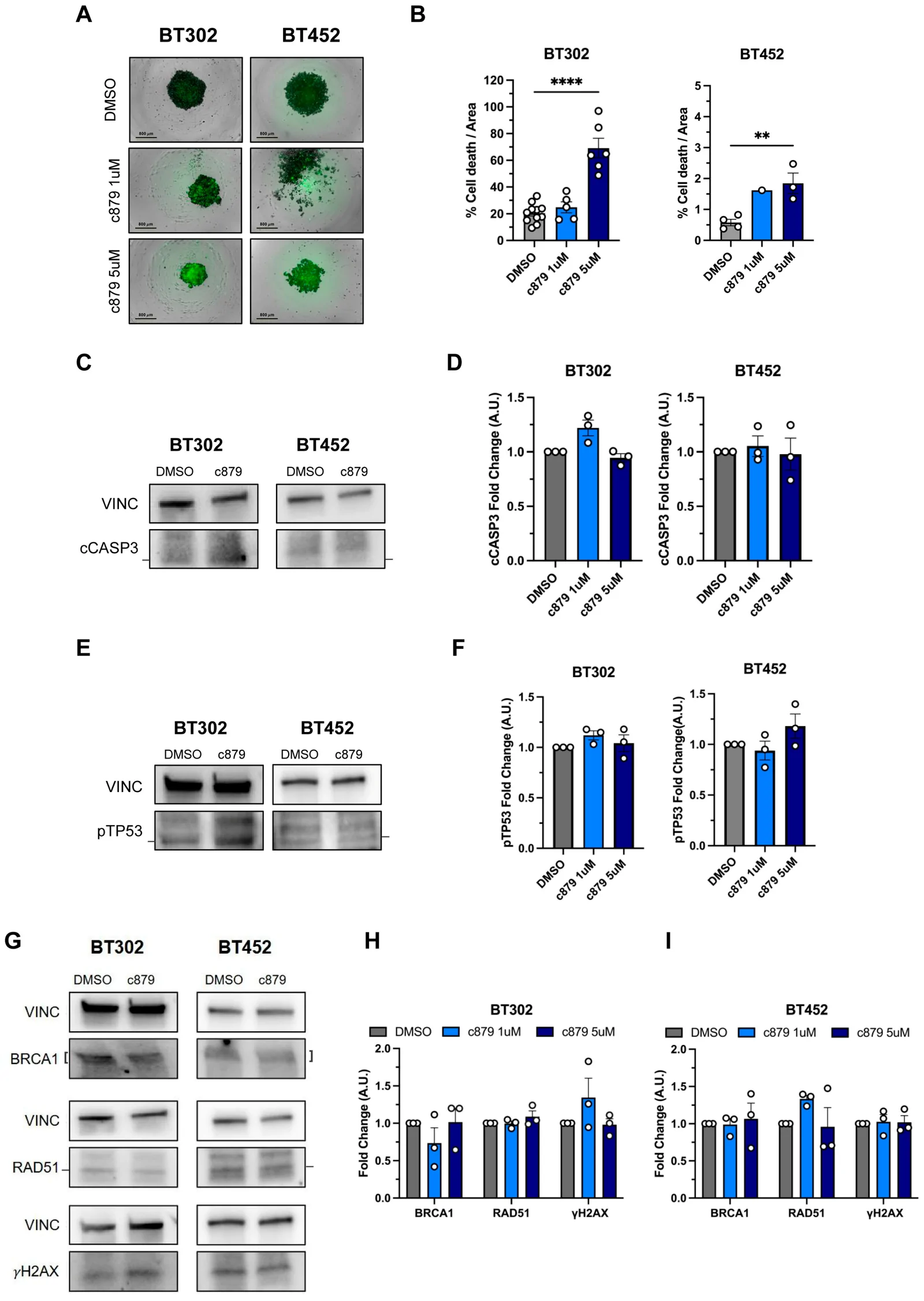
Effects of c879 treatment on cell death and DNA damage response in GSC. (**A**) Exemplar images of BT302 and BT452 spheroids after CellTox^TM^ green cytotoxicity assay at day 7 after treatments. Scale bar: 800 µm. It is noteworthy that, in c879-treated BT452 spheroids, addition of the CellTox^TM^ reagent leads to rapid disgregation. (**B**) Percentage of cell death quantified with green fluorescence intensity and normalized on the spheroid area of BT302 and BT452 at day 7. Each dot indicates an independent biological replicate (BT302 c879 1 µM *n* = 5, 5 µM *n* = 6; BT452 c879 1 µM *n* = 1, 5 µM *n* = 3). At least three spheroids were counted for each treatment condition in each experiment. (**C**,**E**,**G**) Western blot analysis of total lysate from the indicated GSC, 48 h after treatment with DMSO or c879 1 μM. For c879 5 μM see Supplementary Figure S3. Levels of the indicated proteins were analyzed and the internal loading control was vinculin (VINC). (**D**,**F**,**H**,**I**) Quantification of the relative density of cCASP3, pTP53, BRCA1, RAD51, and γH2AX in the indicated GSC. For BRCA1, the position of the quantified doublet is indicated by a bracket. In the other cases in which more bands are visible, the position of quantified bands is indicated by a dash. Each dot indicates an independent biological replicate (*n* = 3). Error bars, SEM. ** *p* < 0.01 **** *p* < 0.0001; paired two-tailed Student’s *t*-test (WB) and unpaired two-tailed Student’s *t*-test (% cell death/area). A.U., arbitrary units.

We next assessed the effects of c879 on cell viability using the CellTox Green cytotoxicity assay. Representative images of BT302 and BT452 spheroids showed increased CellTox-associated fluorescence following c879 treatment (Figure 6A). Quantitative analysis confirmed a significant increase in cytotoxicity in both GSC at 5 µM, whereas treatment with 1 µM produced more limited effects (Figure 6B). A significant reduction in viability, especially at high inhibitor concentrations, was also documented by MTS assay (Supplementary Figure S2B). Western blot analysis of cCASP3 performed 48 h after treatment revealed a modest increase in BT302 cells treated with 1 µM c879, although this increase did not reach statistical significance. At 5 µM, cCASP3 levels were comparable to those of control cells. No significant changes in cCASP3 levels were observed in BT452 cells at either concentration (Figure 6C,D; Supplementary Figure S3A). We next investigated whether c879 treatment affected TP53 signaling and DNA damage-associated pathways. In BT302 cells, pTP53 levels showed a modest increase following treatment with 1 µM c879, although this effect did not reach statistical significance. No detectable changes in pTP53 levels were observed in BT452 cells (Figure 6E,F; Supplementary Figure S3B). Moreover, c879 treatment did not significantly alter BRCA1, RAD51, or γH2AX levels in either cell line (Figure 6G,H; Supplementary Figure S3C). These findings indicate that, under the tested conditions, the selective inhibition of CITK kinase activity may not reproduce the alterations in DNA damage- and HR-associated proteins observed following CITK depletion in BT302 cells.

**Figure 7:**
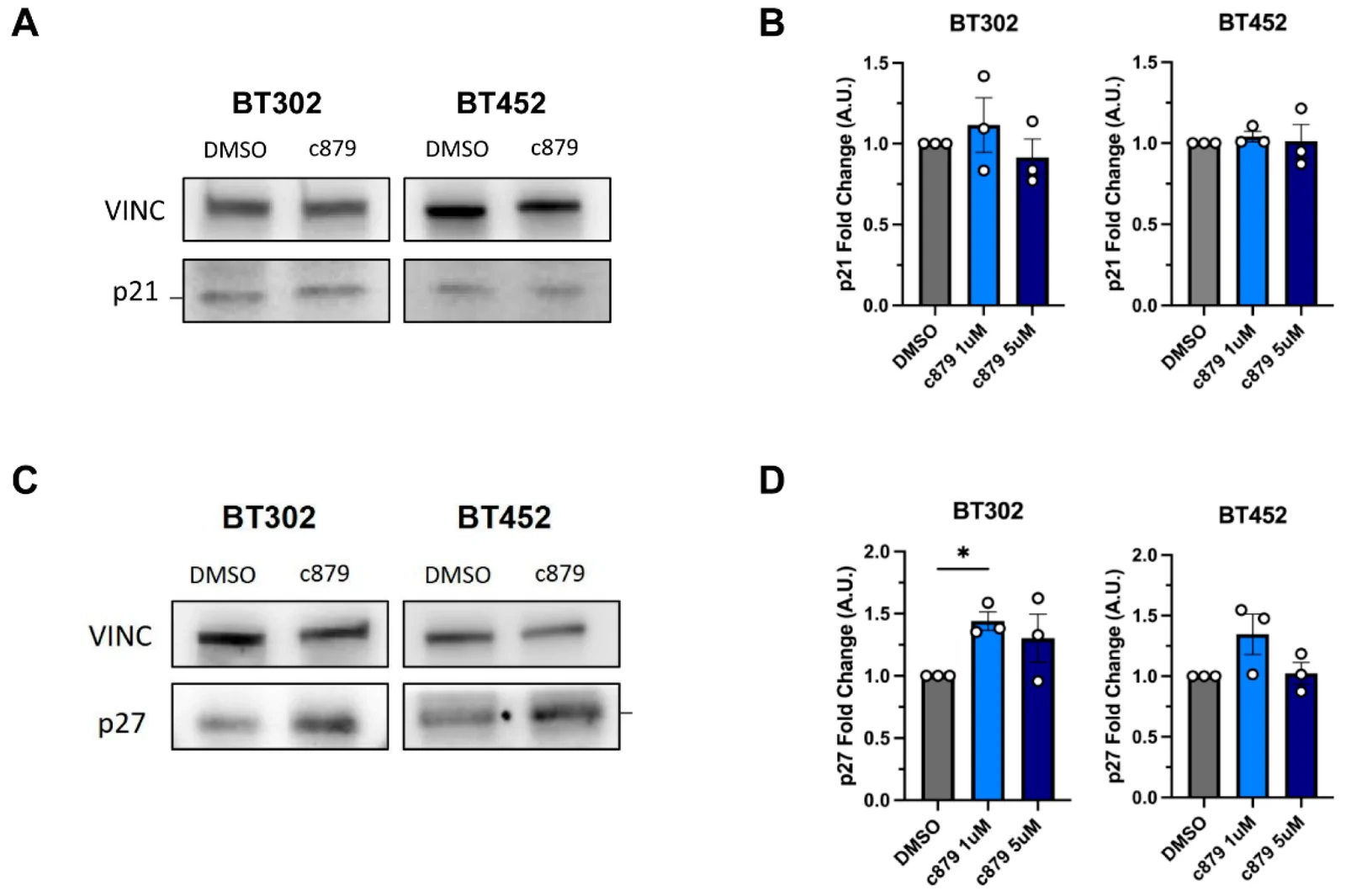
c879 induces p27 expressions in GSC. (**A**,**C**) Western blot analysis of total lysate from the indicated GSC, 48 h after treatment with DMSO or c879 1 µM. For c879 5 μM see Supplementary Figure S3. Levels of the indicated proteins were analyzed and the internal loading control was vinculin (VINC). Where more bands are visible, the position of quantified bands is indicated by a dash. (**B**,**D**) Quantification of the relative density of p21 and p27 in the indicated GSC. Each dot indicates an independent biological replicate (*n* = 3). Error bars, SEM. * *p* < 0.05, paired two-tailed Student’s *t*-test. A.U., arbitrary units.

Given the limited activation of apoptosis following c879 treatment, we next investigated whether c879 could modulate other growth inhibitory responses, such as the expression of the cyclin-dependent kinase inhibitors p21 and p27. Western blot analysis of p21 in both BT302 and BT452 cells showed that c879 treatment did not significantly alter the protein levels at either 1 or 5 µM (Figure 7A,B, Supplementary Figure S3D). In contrast, analysis of p27 revealed an increase following c879 treatment in both GSC, with statistical significance reached only in BT302 cells at 1 µM (Figure 7C,D, Supplementary Figure S3E). Taken together, these results suggest that selective inhibition of CITK kinase activity in GSC may not phenocopy the effects of CITK silencing and may generally produce even weaker phenotypes than the latter treatment.

### 2.6. Lestaurtinib Impairs GSC Spheroids’ Growth and Cytokinesis

**Figure 8:**
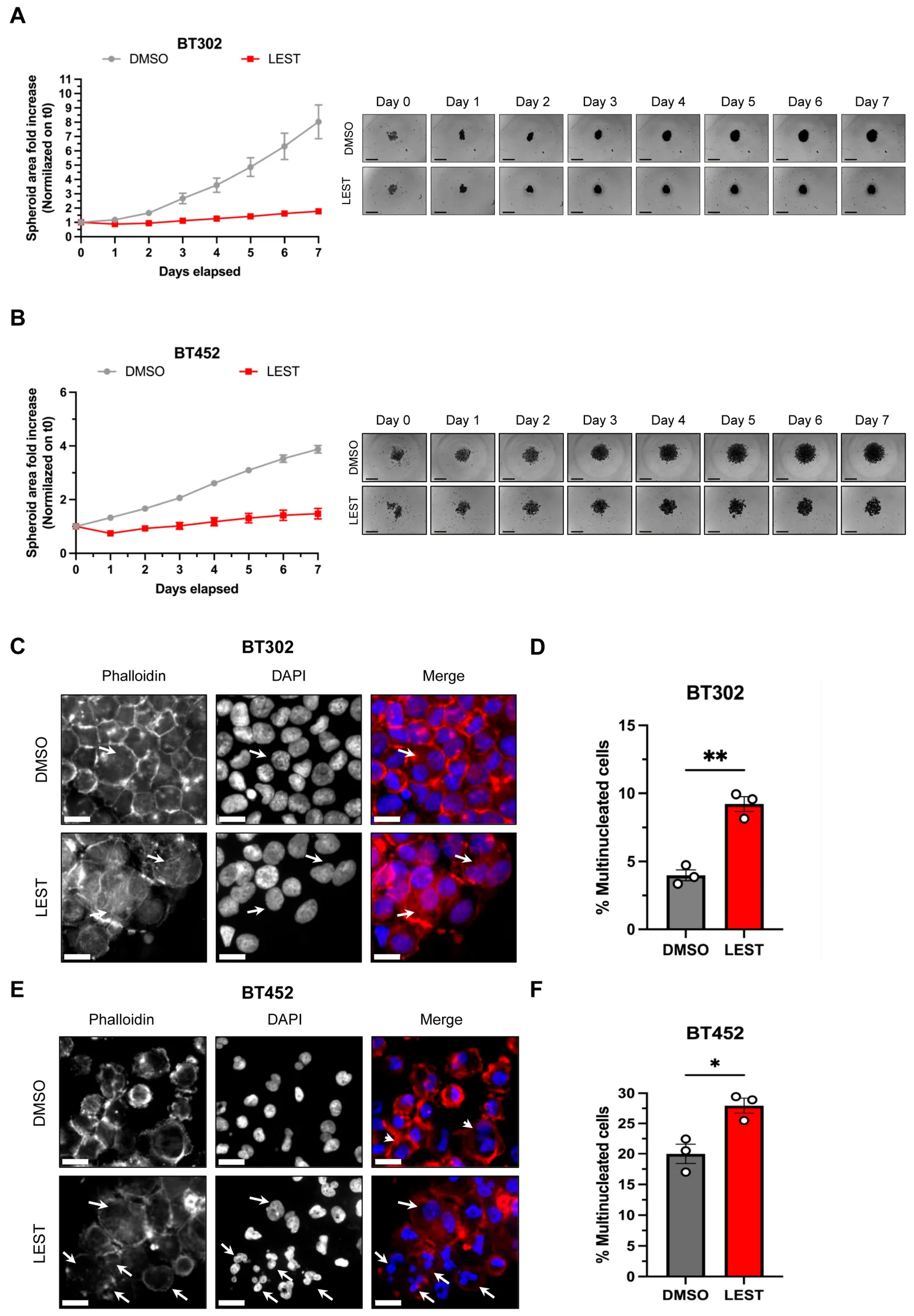
Lestaurtinib decreases proliferation and induces cytokinesis failure in GSC. (**A**,**B**) Cell proliferation assay. Growth curves were obtained by assessing the area of the spheroid, normalized on time 0, at the indicated times after treatment with 100 nM Lestaurtinib (LEST). *n* = 7, with each *n* consisting of three spheroids. The treatment curves were statistically different from control curves at the following levels: BT302 *p* < 0.0001, B452 *p* < 0.0001; two-way ANOVA. Representative images of spheroids. Scale bars: 800 μm. (**C**,**E**) Representative image of BT302 and BT452 cells, respectively, processed for immunofluorescence 48 h after DMSO or LEST treatment and stained with DAPI (blue) and Phalloidin (red). White arrows indicate exemplar multinucleated cells. Scale bars: 20 μm. (**D**,**F**) Quantification of multinucleated BT302 and BT452 cells, respectively, in the indicated treatment, performed as in (**C**,**E**). Each dot indicates an independent biological replicate (*n* = 3). >200 cells were counted for each treatment condition in each experiment. All quantifications were based on at least three independent biological replicates. Error bars, SEM. * *p* < 0.05, ** *p* < 0.01; unpaired two-tailed Student’s *t*-test. A.U., arbitrary units.

To determine whether the effects of the polypharmacological inhibitor Lestaurtinib previously observed in MB cells [19] could also be reproduced in GSC, we treated BT302 and BT452 spheroids with 100 nM Lestaurtinib 24 h after seeding. In both GSC, Lestaurtinib significantly impaired spheroid growth as early as 24 h after treatment. By day 7, spheroid area was reduced by 78% in BT302 cells and 64% in BT452 cells compared with controls (Figure 8A,B). Similar results were obtained even in the multi-spheroid assay (Supplementary Figure S1E). Western blot analysis revealed that Lestaurtinib treatment reduced CITK protein levels in both GSC after 48 h, by 63% in BT302 cells, and by 38% in BT452 cells (Supplementary Figure S4). In addition, Lestaurtinib-treated cells exhibited a significantly increased proportion of multinucleated cells compared with controls in both GSC at 48 h after treatment (Figure 8C–F), consistent with the induction of cytokinesis defects previously observed in MB cells [19].

### 2.7. Lestaurtinib Induces Limited Cytotoxicity and Cell-Line-Specific DNA Damage Responses While Increasing P27 Levels in GSC

**Figure 9:**
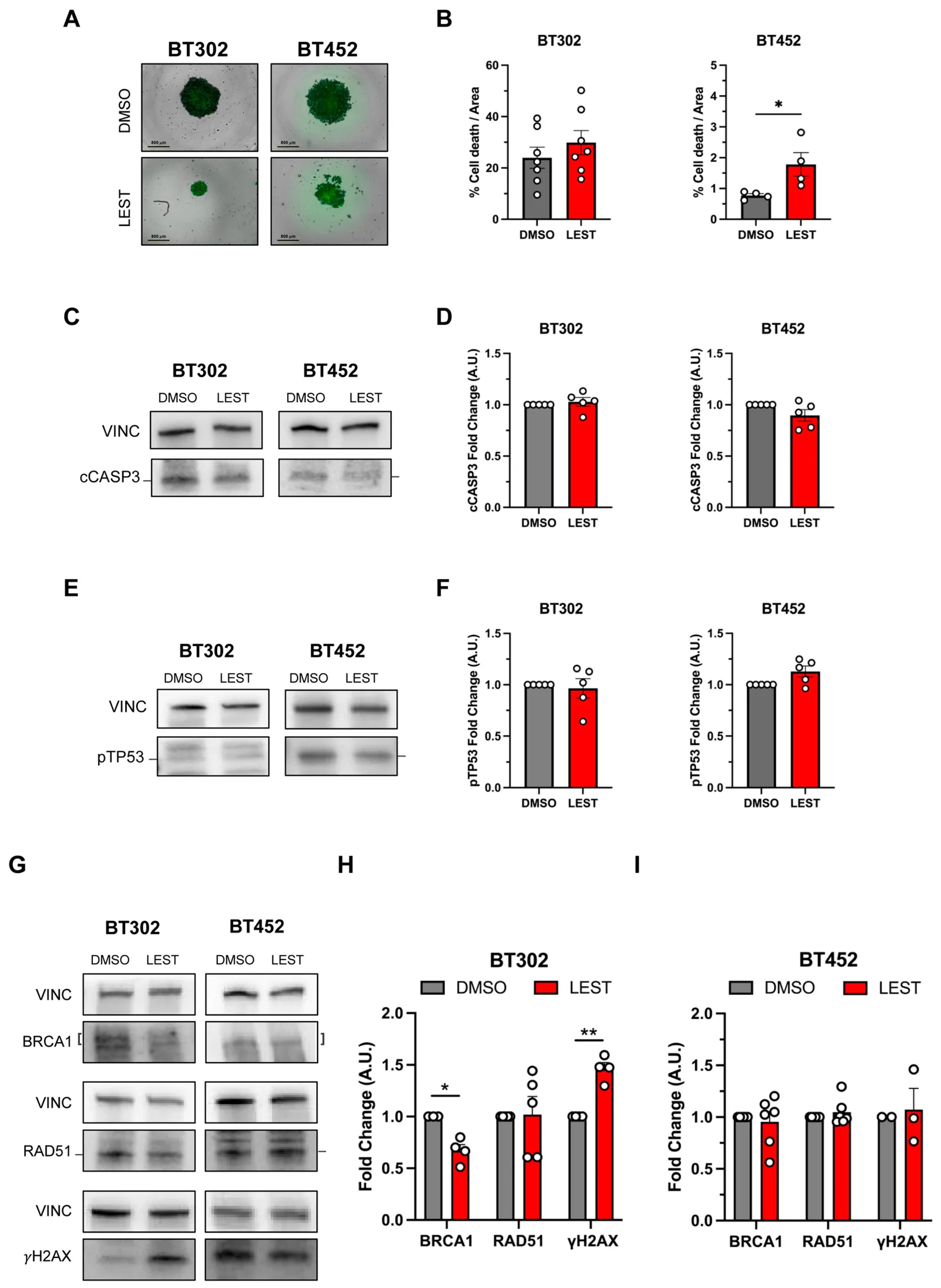
Differential effects of Lestaurtinib on cell death and DNA damage response in GSC. (**A**) Exemplar image of BT302 and BT452 spheroids after CellTox^TM^ green cytotoxicity assay at day 7 after treatment with DMSO or 100 nM Lestaurtinib (LEST). Scale bars: 800 μm. (**B**) Percentage of cell death quantified with green fluorescence intensity and normalized on the spheroid area of BT302 and BT452 at day 7. Each dot indicates an independent biological replicate (BT302 *n* = 8; BT452 *n* = 4). At least three spheroids were counted for each treatment condition in each experiment. (**C**,**E**,**G**) Western blot analysis of total lysate from the indicated GSC, 48 h after treatment with DMSO or LEST. Levels of the indicated proteins were analyzed and the internal loading control was vinculin (VINC). For BRCA1, the position of the quantified doublet is indicated by a bracket. In the other cases in which more bands are visible, the position of quantified bands is indicated by a dash. (**D**,**F**,**H**,**I**) Quantification of the relative density of the indicated proteins in the indicated GSC. Each dot indicates an independent biological replicate (BT302 cCASP3 and pTP53 *n* = 5, BRCA1 and γH2AX *n* = 4, RAD51 *n* = 5; BT452 cCASP3 and pTP53 *n* = 5, BRCA1 and RAD51 *n* = 6, γH2AX *n* = 3). Error bars, SEM. * *p* < 0.05, ** *p* < 0.01; paired two-tailed Student’s *t*-test. A.U., arbitrary units.

We next assessed the effects of Lestaurtinib on cell death using the CellTox Green cytotoxicity assay in individual spheroids (Figure 9A,B). In BT302 cells, Lestaurtinib treatment resulted in a modest increase in cytotoxicity of approximately 10%, although this effect did not reach statistical significance. In contrast, BT452 cells showed a statistically significant increase in cytotoxicity, albeit of limited magnitude. In contrast to the effect produced on MB [19], cCASP3 levels were not significantly altered by Lestaurtinib treatment in either cell line (Figure 9C,D) and pTP53 protein levels were also unchanged in BT302 cells 48 h after treatment, whereas a modest but significant increase was observed in BT452 cells (Figure 9E,F). Notably, MTS assay revealed a consistent decrease of metabolic activity in both cell lines (Supplementary Figure S2E).

We next examined DNA damage- and HR-associated proteins. In BT302 cells, Lestaurtinib treatment increased γH2AX levels and reduced BRCA1 protein levels (Figure 9G–I), consistent with increased DNA damage signaling and a potential impairment of HR-mediated DNA repair. No significant changes in BRCA1, RAD51, or γH2AX levels were detected in BT452 cells. Thus, Lestaurtinib differentially affects DNA damage- and HR-associated proteins in the two GSC, with a more pronounced response in BT302 cells.

**Figure 10:**
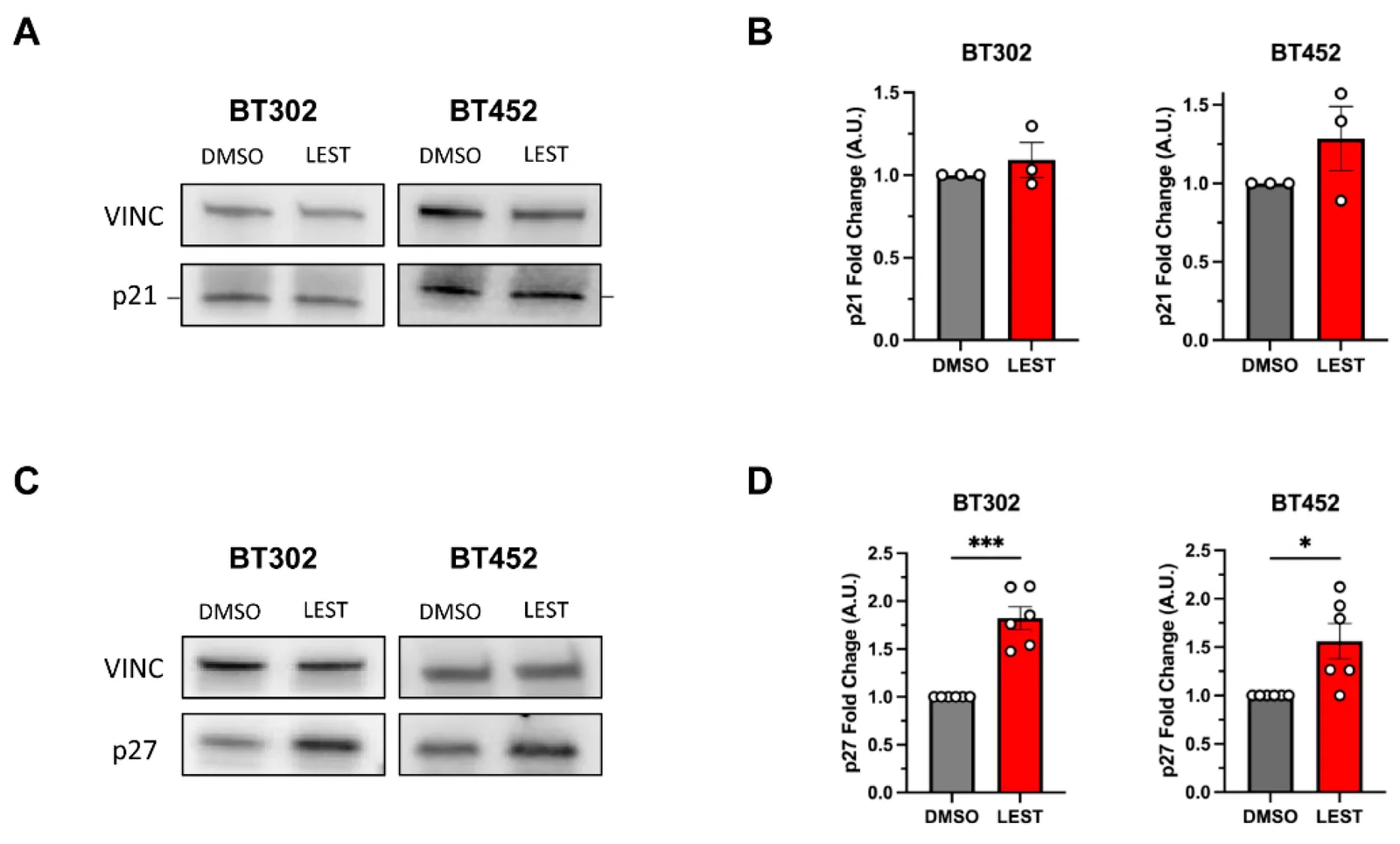
Lestaurtinib consistently induces p27 expressions in GSC. (**A**,**C**) Western blot analysis of total lysate from the indicated GSC, 48 h after treatment with DMSO or 100 nM Lestaurtinib. The levels of p21 and p27 were analyzed and the internal loading control was vinculin (VINC). For p21, the position of the quantified band is indicated by a dash. (**B**,**D**) Quantification of the relative density of p21 and p27 in the indicated GSC. Each dot indicates an independent biological replicate (p21 *n* = 3; p27 *n* = 6). Error bars, SEM. * *p* < 0.05, *** *p* < 0.001; paired two-tailed Student’s *t*-test. A.U., arbitrary units.

Finally, we examined the cell-cycle regulators p21 and p27. Lestaurtinib did not significantly alter p21 levels in BT302 cells and induced only a modest, non-significant increase in BT452 cells (Figure 10A,B). In contrast, p27 levels were significantly increased in both GSC (Figure 10C,D). Together, these findings indicate that Lestaurtinib strongly inhibits GSC spheroid growth despite inducing only limited cytotoxicity and no detectable increase in cCASP3, suggesting that its antiproliferative effects may involve other mechanisms than robust induction of canonical apoptosis, potentially including p27-associated cell-cycle regulation and decreased metabolic activity.

## 3. Discussion

In this study, we investigated whether GSCs share the vulnerability to CITK loss previously identified in MB cells [17,18,19], and whether this can eventually be reproduced by selective or poly-pharmacological inhibitors of CITK kinase activity. Our results show that CITK depletion may impair the growth of patient-derived GSC but also reveal substantial differences from MB in the homogeneity of cellular and molecular responses. Across the different experimental conditions, spheroid growth inhibition was more consistent than DNA damage or apoptosis. Increases in p27 levels were also observed under the three different treatments, raising the possibility that p27 may be a common player in responses induced by all of them.

The cellular response to CITK depletion in GSCs differed in several respects from that previously described in MB cells [17,18]. Although CITK depletion increased the frequency of multinucleated cells, the cytokinesis defect was relatively modest (Figure 1C–F). Moreover, both GSCs displayed substantial basal levels of multinucleation, suggesting that the additional contribution of CITK knockdown to this phenotype may be limited. These observations suggest that cytokinesis failure alone is unlikely to account for the spheroid growth inhibition observed in these GBM models.

The effects of CITK depletion on genome integrity may be similarly heterogeneous (Figure 2A–C). In BT302 cells, CITK depletion increased γH2AX levels and reduced BRCA1 and RAD51 levels, consistent with increased DNA damage signaling and a potential impairment of HR-mediated DNA repair. In contrast, these changes were weak or absent in BT452 cells, a result that may be correlated with the TP53 mutant status of this cell line. Thus, the DNA damage-associated response to CITK depletion appears less pronounced and more heterogeneous in GBM than previously observed in MB [17,18]. The different responses of BT302 and BT452 indicate that the consequences of CITK depletion may be strongly influenced by cell-intrinsic context, including TP53 status, which may modulate the DNA damage response elicited by CITK loss.

In both GBM models, growth inhibition was accompanied by only limited evidence of canonical apoptosis. Cleaved Caspase-3 levels showed only modest changes, and pTP53 and p21 induction were limited (Figure 3 and Figure 4). These observations argue against a major contribution of the canonical TP53–p21 response to the growth inhibition induced by CITK depletion [17,18], even in presence of wild type TP53. In contrast, p27 levels were markedly increased in both GSC. An increase in p27 was also a prominent feature of the response to CITK loss previously observed in MB [17], suggesting that p27-dependent responses may contribute to the antiproliferative effects of CITK depletion.

The increase in p27 levels is also of potential mechanistic interest, because p27 can exert cyclin-dependent kinase (CDK)-independent functions, including a role in the regulation of cytokinesis that is mediated, at least in part, through its interaction with CITK [38,39]. Nevertheless, the relatively modest cytokinesis phenotype observed in both GSCs makes it unlikely that p27 accumulation can be explained solely because of cytokinesis failure. It would therefore be interesting to determine the mechanism responsible for p27 accumulation following CITK depletion and whether p27 is functionally required for spheroid growth inhibition.

Experiments with the selective CITK inhibitor c879 provided an opportunity to distinguish the consequences of CITK protein depletion from those associated with inhibition of its catalytic activity. Notably, GSC cells appear to differ from the tumor cell lines previously tested with c879, in which no significant antiproliferative activity was reported [27]. In our GBM models, c879 reduced spheroid growth and increased cytotoxicity, demonstrating that these cells may be responsive to selective pharmacological inhibition of CITK (Figure 5). However, c879 reproduced only a subset of the cellular and molecular effects observed following CITK depletion. Its effects on multinucleation were relatively modest, and it did not reproduce the alterations in γH2AX, BRCA1, and RAD51 observed following CITK depletion in BT302 cells. Similarly, changes in p27 were restricted to BT302 cells under selected treatment conditions. Thus, selective inhibition of CITK kinase activity may only partially recapitulate the phenotype associated with CITK depletion.

Our findings suggest that inhibition of CITK catalytic activity alone would be most likely insufficient to reproduce the full antiproliferative consequences of CITK depletion. Indeed, it is not unusual for multi-domain proteins that inhibit only catalytic function and do not recapitulate the effect of degradation of the entire protein [40,41]. Several non- mutually exclusive explanations remain possible. The pharmacological inhibition achieved under our experimental conditions may be incomplete, or the cellular activity of c879 may be limited in these models. Alternatively, non-catalytic functions of CITK may contribute to the phenotype produced by protein depletion [14]. In this context, targeted protein degradation may represent a more suitable pharmacological strategy than kinase inhibition. Notably, in recent years, Proteolysis-Targeting Chimeras (PROTACs) became an attractive therapeutic strategy against different molecular targets, due to their capability to induce complete protein degradation [42]. This may represent an interesting approach to develop a novel approach for CITK inhibition in sensitive tumors.

The polypharmacological inhibitor Lestaurtinib produced a substantially stronger reduction in spheroid growth and a more pronounced cytokinesis defect in both GBM models. Lestaurtinib also reduced CITK protein levels, although the extent of this reduction differed between BT302 and BT452 cells. Nevertheless, the molecular response did not fully phenocopy that previously observed in MB cells [19]. In BT302 cells, Lestaurtinib increased γH2AX and reduced BRCA1 levels, whereas these responses were essentially absent in BT452 cells. Moreover, despite its pronounced effect on spheroid growth, Lestaurtinib induced only limited cytotoxicity and no detectable increase in cCASP3 in either cell line. In contrast, p27 levels were significantly increased in both GBM models. Thus, as observed following CITK depletion, the effects of Lestaurtinib on GSC may involve variable levels of growth inhibition without robust activation of canonical apoptosis.

Because Lestaurtinib inhibits multiple kinases [43], its stronger antiproliferative activity cannot be attributed specifically to CITK inhibition. Rather, it may reflect the combined perturbation of several pathways involved in GSC proliferation and survival. The partial overlap between the effects of Lestaurtinib and CITK depletion—including cytokinesis defects, p27 accumulation, and, in BT302 cells, alterations in DNA damage-associated proteins—is noteworthy but does not establish CITK as the principal mediator of Lestaurtinib activity. Identifying the pathways responsible for its effects in GSC will therefore require further investigation.

The different responses of BT302 and BT452 further emphasize the strong influence of cellular context on the consequences of CITK perturbation. A major limitation of the present study is the use of only two patient-derived GBM models, which precludes assessment of the extent to which these responses are representative of the broader molecular heterogeneity of GBM. In particular, the DNA damage-associated response differed markedly between the two models, despite growth inhibition being observed in both. Analysis of a larger and molecularly diverse panel of patient-derived GBM models will therefore be required to identify determinants of sensitivity to CITK depletion and pharmacological inhibition and to establish whether specific molecular features predict the cellular response.

Overall, our findings suggest that CITK may not be a very significant vulnerability in patient-derived GSCs and reveal important differences between CITK depletion and selective inhibition of its kinase activity. The partial phenotypic overlap among CITK knockdown, c879, and Lestaurtinib suggests that catalytic and non-catalytic functions of CITK, as well as CITK-independent effects of polypharmacological inhibition, may contribute to the observed responses. At the same time, the heterogeneity observed between the two GBM models indicates that the consequences of CITK perturbation are strongly context dependent. Defining the mechanisms underlying this heterogeneity, including the functional relevance of p27 accumulation, and extending these analyses to a broader panel of patient-derived models will be essential for establishing whether and how CITK can be effectively exploited as a therapeutic target in GBM in at least a subset of cases, eventually in combination with the present standard of care treatments, i.e., ionizing radiation and Temolzolomide, or with other DNA damaging drugs.

## 4. Materials and Methods

### 4.1. Cell Culture

BT302 and BT452 GSC were derived in 2009 and 2011, respectively, from patients who underwent surgical resection of glioblastoma at the Fondazione IRCCS Istituto Neurologico C. Besta (Milan, Italy), in accordance with the guidelines of the institutional Ethics Committee. Written informed consent was obtained from all patients, and the study was conducted in accordance with the Declaration of Helsinki.

Both neurosphere lines were cryopreserved at passage 15 in complete medium supplemented with 10% DMSO. Cells were initially maintained at −80 °C for 24 h and subsequently transferred to liquid nitrogen for long-term storage. All experiments were performed using cells originating from the same frozen cell bank. For each experimental series, an aliquot was thawed once, expanded in culture, and used at passages 17–18, with no additional freeze–thaw cycles. BT302 and BT452 cells were used within this passage range for all assays described in this study. These GSCs were previously described [6,28] (for details on genetic characterization see Supplementary Table S1) and cultured in DMEM/F12 15 mM HEPES (Gibco, Waltham, MA, USA) supplemented with B27 (Gibco, Waltham, MA, USA), PSF (Gibco, Waltham, MA, USA), Glutamine (Gibco, Waltham, MA, USA), Heparin 25 mg/mL (Sigma-Aldrich, St. Louis, MO, USA), EGF (Sigma-Aldrich, St. Louis, MO), and FGF (PeproTech, part of Thermo Fisher Scientific, Cranbury, NJ, USA) 2 ng/mL. Cells were grown at 37 °C, in a humidified incubator, with 5% CO_2_ and routinely tested for mycoplasma contamination with the following oligonucleotide sequences:

MYCO1: 5′-ACTCCTACGGGAGGCAGCAGTA-3′;

MYCO2: 5′-TGCACCATCTGTCACTCTGTTAACCTC-3′.

### 4.2. Transfection for RNAi

Transfection for RNAi was performed as previously described [17] with published CITK double-stranded RNA [13]. Non-targeting pool (Dharmacon, Lafayette, CO, USA) was used as a negative control. GSCs were plated on six-well plates, transfected using 6.25 μL of the required siRNA (20 μM) together with 5 μL of Lipofectamine 3000 (Invitrogen, Carlsbad, CA, USA).

### 4.3. Analysis of GSC Spheroid Growth

To analyze GSC single spheroid growth, BT302 or BT452 cells were seeded into 96-well ultra-low attachment round bottom plates (*n* = 2500 cells/well), allowing spontaneous aggregation and the formation of a single spheroid per well. Treatments were performed either at the time of cell seeding for siRNA transfection experiments, or 24 h post-seeding for inhibitor treatments. GSC proliferation was monitored using the Incucyte^®^ SX5 Live-Cell Analysis System, software v2025c (Sartorius, Goettingen, Germany). The system automatically acquired phase-contrast images at defined time intervals (24 h) for 7 days, enabling continuous, non-invasive monitoring of spheroid growth over time. For growth curve analysis, spheroid growth was normalized to the spheroid area measured at the initial time point, and the relative growth was calculated over time.

To analyze GSC multi-spheroid growth, BT302 or BT452 cells were seeded into 96-well flat bottom plates (*n* = 10,000 cells/well), allowing spontaneous aggregation and the formation of multi spheroid per well. Treatments were performed either at the time of cell seeding for siRNA transfection experiments, or 24 h post-seeding for inhibitor treatments. GSC proliferation was monitored using the same Incucyte^®^ Live-Cell Analysis System as above. Phase-contrast images were acquired at day 3 after treatment, and the instrument automatically detected the spheroid mask, allowing the average spheroid area to be calculated for each well. Data was expressed as a percentage relative to the untreated control, which was set at 100%.

### 4.4. CellTox^TM^ Green Cytotoxicity Assay

To measure cytotoxicity, CellTox^TM^ Green Reagent (Promega Corporation, Madison, WI, USA) was added to GSC spheroids at the end point of single spheroid growth experiments, according to the manufacturer’s instructions. Fluorescence was recorded using the same Incucyte^®^ Analysis System as above. The fluorescence area was normalized to the total spheroid area.

### 4.5. Compounds

The polypharmacological inhibitor Lestaurtinib was purchased from Bio-Techne (Tocris Biotechne group, Minneapolis, MN, USA). The highly selective CITK inhibitor C3TD879 (also referred to as c879) was kindly provided by the group of Shaun R. Stauffer at the Cleveland Clinic (Cleveland, OH, USA).

### 4.6. Antibodies

The following antibodies were used: mouse monoclonal anti-citron (#611377) and mouse monoclonal anti-p27 (#610242) from Transduction Laboratories, BD Biosciences (Franklin Lakes, NJ, USA); mouse monoclonal anti-vinculin (#V9131) from Sigma-Aldrich; rabbit polyclonal anti-cleaved Caspase 3 (#9661S), anti-TP53 phospho Ser15 (#9284S), and anti-γH2AX (S139; 20E3; #2577) from Abcam (Cambridge, UK); and rabbit monoclonal anti-RAD51 (#sc-8349), mouse monoclonal anti-BRCA1 (#sc-6954), and rabbit polyclonal anti-p21 (#sc-756) from Santa Cruz (Dallas, TX, USA).

### 4.7. Western Blotting

For Western blot, cells were seeded into 6-well, lysed in RIPA buffer (1% NP40, 150 mmol/L of NaCl, 50 mmol/L of Tris-HCl pH 8, 5 mmol/L of EDTA, 0.01% SDS, 0.005% sodium deoxycholate, Roche protease inhibitors and PMSF) for 10 min at 4 °C and centrifuged at 13,000 rpm at 4 °C for 10 min. For immunoblots, equal amounts of each sample were resolved by SDS–PAGE and blotted to nitrocellulose membrane (Bio-Rad Laboratories, Hercules, CA, USA). Each protein was normalized to its corresponding vinculin level. Nevertheless, it must be underscored that Western blotting remains a semi-quantitative method for measuring protein levels.

### 4.8. Immunofluorescence

Cells were first spun in Thermo Scientific Cytospin 4 (Thermo Fisher Scientific, Waltham, MA, USA) at 500 rpm, for 10 min, and then processed for immunofluorescence, as previously published [18]. Cells were counterstained with 0.5 µg/mL of DAPI and phalloidin for 30 min and washed with PBS. Finally, cell slides were mounted with Prolong (Thermo Fisher Scientific, Waltham, MA, USA).

### 4.9. MTS-Assay

Cell viability was assessed using the CellTiter 96^®^ AQueous One Solution Cell Proliferation Assay (MTS; Promega, G3580), according to the manufacturer’s instructions. Multi-spheroids were generated as described in the “Proliferation analysis” section and treated as indicated. At day 3 post-treatment, the MTS assay was performed and absorbance was measured with Infinite^®^ M Nano (Tecan, Grödig/Salzburg, Austria) to assess cell viability.

### 4.10. Software

WB protein band intensity was analyzed using ImageLab 6.1 Software. Proliferation and green fluorescent area (cell death) were recorded using the Incucyte2026A program. Immunofluorescence images were acquired using Confocal LSM 980 Airyscan 2 (ZEISS, Jena, Germany) and analyzed using ZeissZEN 3.9 software. All graphs were realized using GraphPad Prism Version 9.5.1.

### 4.11. Statistical Analysis

Statistical analyses were performed using Microsoft Office Excel (Version 16, Microsoft Corporation, Redmond, WA, USA) and GraphPad (Version 9, GraphPad Software, San Diego, CA, USA). Paired two-tail Student’s *t*-test was used for all WB analysis. A two-way ANOVA test was used for cell growth curves. An unpaired two-tail Student’s *t*-test was used if not otherwise specified. Data are shown as the mean values of at least three independent experiments and standard error of the mean (mean ± SEM).

## Data Availability

The original contributions presented in this study are included in the article/Supplementary Material. Further inquiries can be directed to the corresponding author.

## Supplementary Materials

The following supporting information can be downloaded at https://www.mdpi.com/article/10.3390/ijms27219678/s1.

## Abbreviations

The following abbreviations are used in this manuscript:

CITK	Citron kinase
CNS	Central nervous system
DSBs	Double-strand breaks
GBM	Glioblastoma multiforme
GSC	Glioblastoma stem cells
HR	Homologous recombination
MB	Medulloblastoma
MCPH	Primary congenital hereditary microcephaly
