# Supplementary material for: Exploration of Alternative Approaches to Citron Kinase Inhibition for Glioblastoma Treatment
Source: Int J Mol Sci. 2026 Oct 29;27(21):9678. doi: 10.3390/ijms27219678 (PMC13763391; doi:10.3390/ijms27219678)
Supplement: Supplementary file 1 [file ijms-27-09678-s001.zip › ijms-4571545-supplementary.pdf]

Supplementary table and figures

Table S1

	BT302	BT452
**Wanq Q. Subtype PMID: 29316430**	Mesenchymal	Mesenchymal
**Wanq Z. Subtype PMID: 32649888**	non I-II	non I-II
**IDH1 status**	wt	wt
**TERT promoter status**	c.1-146C>T	c.1-124C>T
**TP53 status**	wt	p.G245D
**PTEN status**	p.R130Q	p.R130Efs*4
**CDKN2A copies**	0	0
**PDGFRA copies**	15,54	3,04
**EGFR copies**	6,91	42,59
**HGF copies**	8,24	6,10
**MET copies**	6,73	3,58
**FGFR3 copies**	0,92	2,80
**MYC copies**	1,199	2,570
**PIK3CA copies**	2,20	2,29
**CDK4 copies**	1,67	2,10
**CDK6 copies**	2,29	2,58
**MDM2 copies**	2,06	1,83
**MDM4 copies**	3,02	3,54

Table S1. Characterization of BT302 and BT452 cells [29].

Figure S1

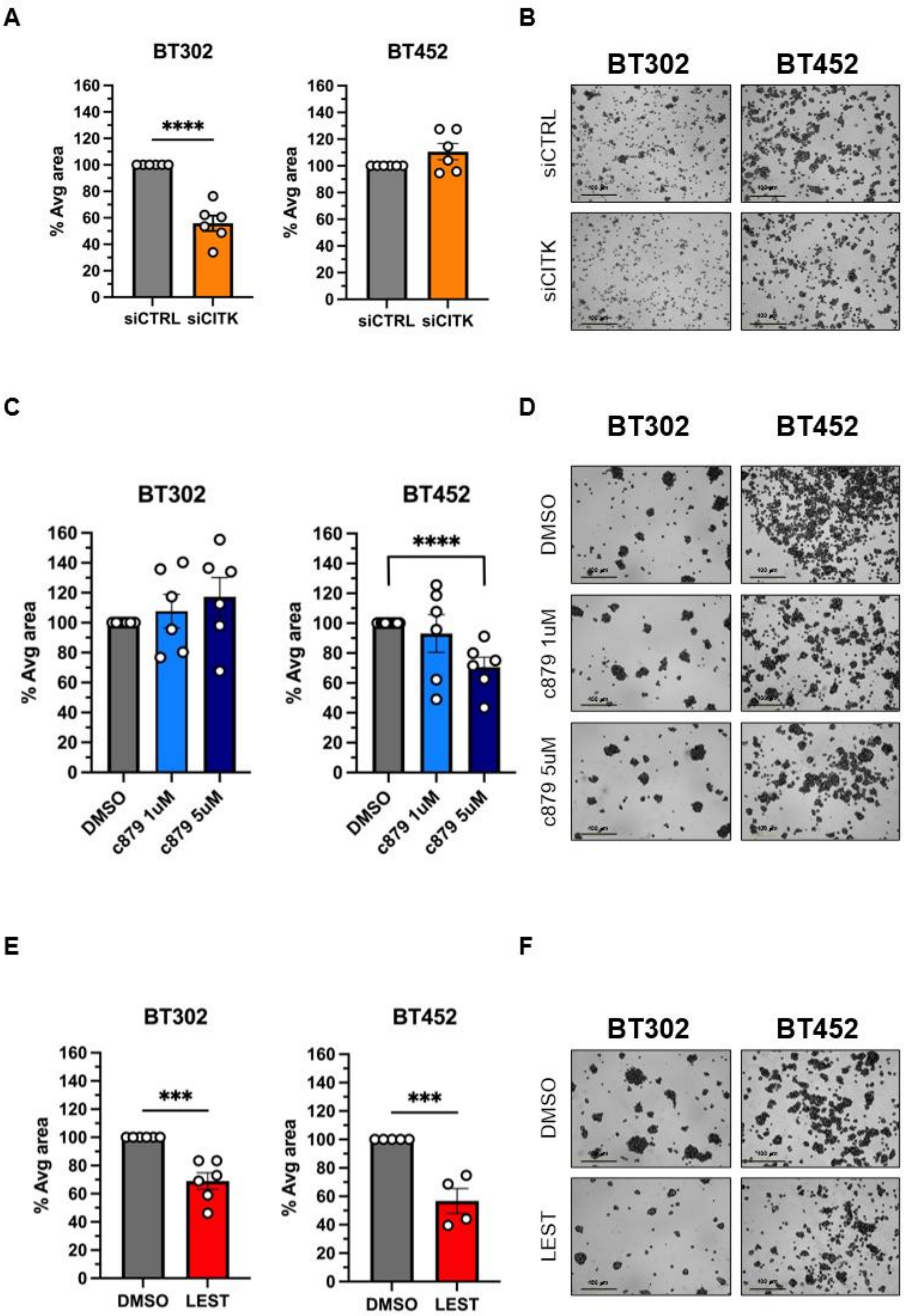

Figure S1. *Effect of the indicated treatments on GSC multi spheroids growth.*

A) Multi spheroid average area in BT302 and BT452 cells treated with control (siCTRL) or CITK-specific siRNAs (siCITK), at day 3 after transfection. Values are expressed as a percentage relative to the average area of siCTRL-treated cells. Each dot represents an independent biological replicate (BT302 n = 6; BT452 n = 6). At least three well were analyzed for each treatment condition in each experiment. (C) Multi spheroid average area in BT302 and BT452 cells treated with vehicle (DMSO) or with the indicated concentrations of c879, at day 3 after treatment addition. Each dot represents an independent biological replicate (BT302 n = 6; BT452 n = 6). At least three well were analyzed for each treatment condition in each experiment. (E) Multi spheroid average area in BT302 and BT452 cells treated with vehicle (DMSO) or with Lestaurtinib, at day 3 after treatment addition. Each dot represents an independent biological replicate (BT302 n = 6; BT452 n = 4). At least three well were analyzed for each treatment condition in each experiment. (B, D, F) Exemplar image of BT302 and BT452 multispheroids at day 3 after treatments. Scale bars = 400 µm. Error bars, SEM. *P<0.05, **P<0.01, ***P<0.001; unpaired two-tailed Student's t-test

Figure S2

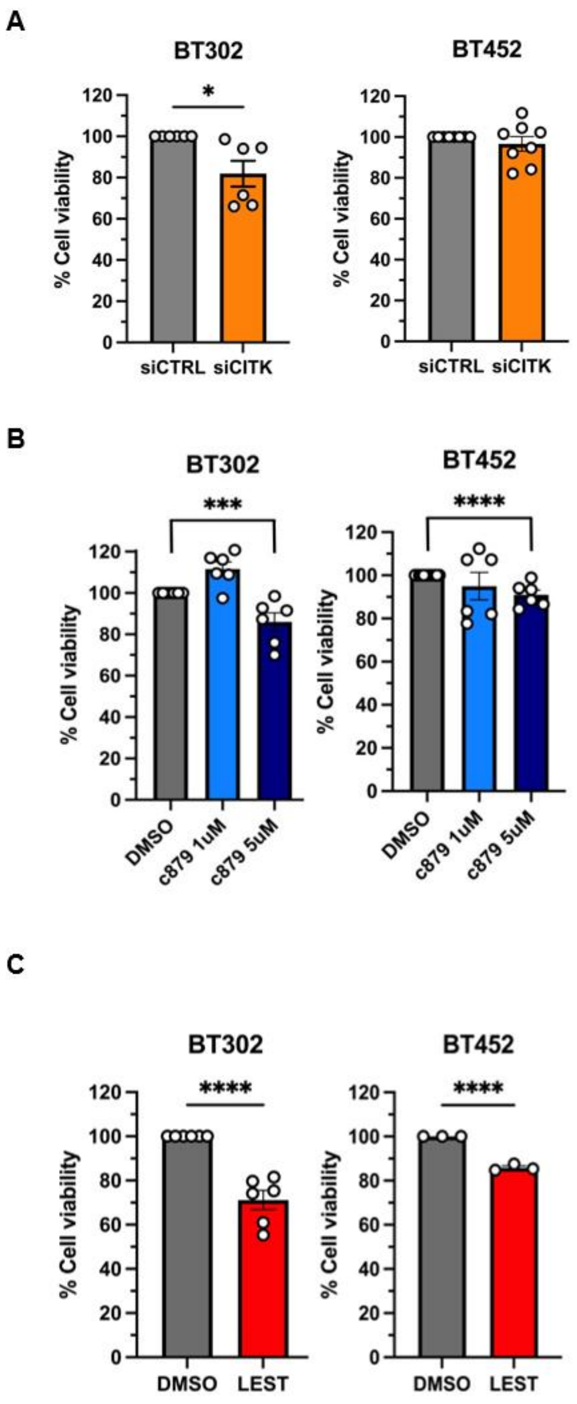

Figure S2. *Effect of the indicated treatments on GSC viability.*

(A, B, C) Cell viability of BT302 and BT452 GSC, after 3 days of treatment with CITK-specific siRNAs (A), with the indicated concentrations of c879 (B) and with Lestaurtinib (C), measured by MTS assay 3 days after the beginning of the different treatments. Values are expressed as a percentage relative to the corresponding control conditions. Each dot represents an independent biological replicate (BT302 n=6; BT452 siCITK n=8, c879 n=6, LEST n=3). At least three well were analyzed for each treatment condition in each experiment. Error bars, SEM. *P<0.05, **P<0.01, ***P<0.001; unpaired two-tailed Student's t-test.

Figure S3

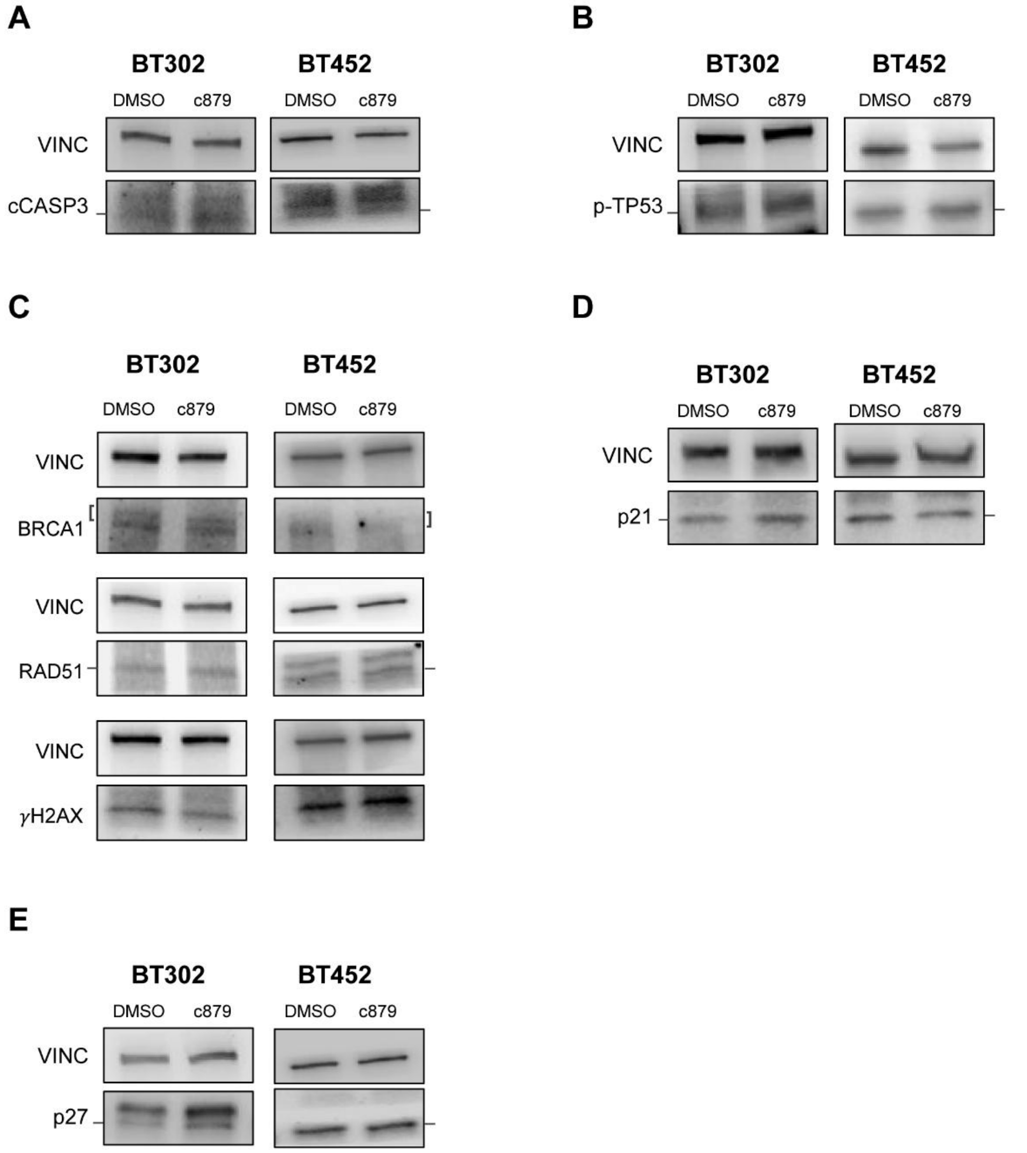

Figure S3. *Western blot analysis of GSC cells treated with 5uM c879.*
(A-E) Western blot analysis of total lysate from the indicated GSC, 48 hours after treatment with DMSO or c879 5μM. Levels of the indicated proteins were analyzed and the internal loading control was vinculin (VINC). Quantifications of specific bands are reported in Fig. 7. For BRCA1, the position of the quantified doublet is indicated by a bracket. In the other cases in which more bands are visible, the position of quantified bands is indicated by a dash.

Figure S4

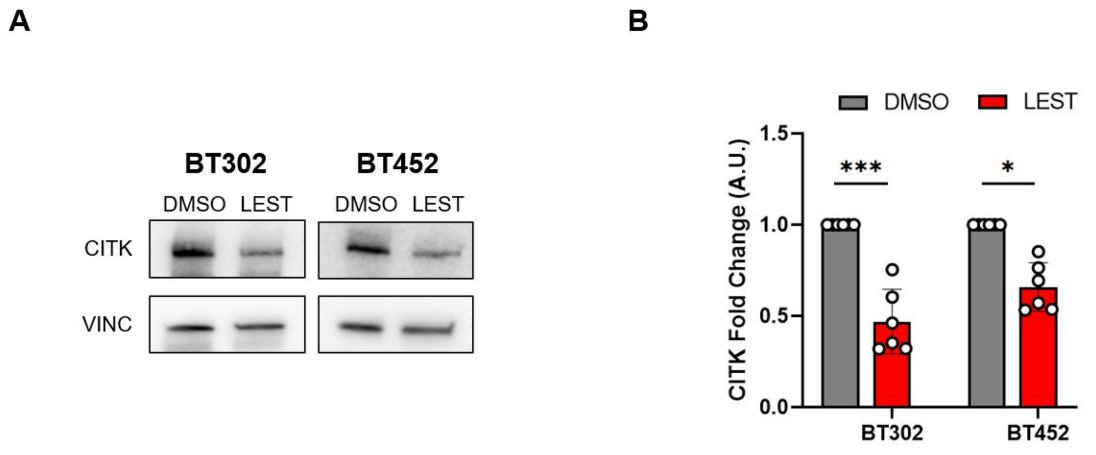

Figure S4. *Lestaurtinib alters CITK protein levels.*

(A) Western blot analysis of total lysate from the indicated cell lines, 48 hours after treatment with (A) Western blot analysis of total lysate from the indicated lines 48 hours after treatment with DMSO or 100 nM Lestaurtinib. The internal control was vinculin (VINC). (B) Quantification of the relative density of CITK in treated cells. Each dot indicates an independent biological replicate. All quantifications were based on at least three independent biological replicates. Error bars, SEM. **P<0.01 ***P<0.01; unpaired two-tailed Student's t-test.
